# Supplementary material for: Behaviour Change Techniques Used in Mediterranean Diet Interventions for Older Adults: A Systematic Scoping Review
Source: Nutrients. 2023 Feb 27;15(5):1189. doi: 10.3390/nu15051189 (PMC10005068; doi:10.3390/nu15051189)
Supplement: Supplementary file 1 [file nutrients-15-01189-s001.zip › Nutrients_Table S2_Study characteristics.pdf]

Table S2. Characteristics of included studies

Author, (year), country	Study design	Target group/sample/health condition	Study aim	Intervention duration	Longitudinal follow-up duration[a]	Sample size, retention at longest follow-up; age: mean ± SD or mean/median [range]; sex: n (%); BMI: mean ± SD or mean/median [range]	Outcome(s) and measure(s)	Intervention effect compared to baseline
Alonso-Dominiguez et al. (2019), Spain	RCT, parallel	T2D	To assess the effectiveness of a multifactorial intervention for improving adherence to the MD and diet quality among adults with T2D	3 months	9 months (12 months since baseline)	Sample size: n = 204 (Intervention n = 102, Control n = 102). Retention: Intervention 92.2%, Control 89.2%. Age: Intervention 60.8 ± 7.8, Control 60.4 ± 8.4. Sex: Intervention 52F (51.1%), Control 41F (40.2%). BMI: Intervention 29.5 ± 4.2, Control 30.3 ± 5.6.	MEDAS, weight, waist circumference, BMI, TC, LDL-c, HDL-c	Post intervention:<br>↑ MEDAS (p<0.001)<br>↑ EVOO ≥4tbsp/day (p<0.001)<br>↑ vegetables ≥2s/d (p<0.001)<br>↑ fruits ≥3s/d (p=0.004)<br>↑ fish/seafood ≥3s/w (p=0.018)<br>↑ nuts ≥3s/w (p<0.001)<br>↑ white meat more than red meat (p<0.001)<br>↑ sofrito sauce ≥2s/w (p<0.001)<br>↓ commercial bakery products ≤s/w (p=0.003)<br>↓ waist circumference (p<0.001)<br><br>Follow-up:<br>↑ MEDAS (p<0.001)<br>↑ EVOO ≥4tbsp/day (p<0.001)

								↑ nuts ≥3s/w (p<0.001)<br>↑ white meat more than red meat (p=0.004)<br>↑ sofrito sauce ≥2s/w (p=0.004)
Baguley et al. (2021), Australia	RCT, parallel	Men with prostate cancer	To determine the effects of the MD on cancer-related fatigue and quality of life in men with androgen deprivation therapy treated prostate cancer	3 months	N/A	Sample size: n = 23 (Intervention n = 12, Control n = 11). Retention: Intervention 91.6%, Control 90.9%. Age: Intervention 66.6 ± 7.6, Control 65.1 ± 7.9. BMI: Intervention 27.4 ± 3.4, Control 30.6 ± 2.9.	MEDAS, dietary intake (WDI), IL-6/8, weight, body fat percentage	↑ MEDAS<br>↑ vegetables (p<0.001)<br>↑ PUFA (p=0.043)<br>↑ omega-3 fats (p=0.033)<br>↑ fibre (p<0.001)<br>↓ energy intake (p=0.032)<br>↓ processed meat s/d (p<0.001)<br>↓ dairy s/d (p=0.033)<br>↓ SFA (p<0.001)<br>↓ weight (p<0.001)<br>↓ fat mass (p=0.032)<br>≠ IL-6/8
Berendsen et al. (2018), multi-country (Italy, Poland, France, Netherlands, UK)	RCT, parallel	Healthy older adults	To evaluate if a personally tailored Mediterranean-like dietary pattern is effective for shifting dietary intake	12 months	N/A	Sample size: n = 1294 (Intervention n = 644, Control n = 650). Retention: 80.4% (Intervention 88.7%, Control 87.7%). Age: Intervention 70.7 ± 4.0, Control 71.1 ± 3.9. Sex: Intervention 248M (43%), Control 26M (46%). BMI: Intervention 26.7 ± 4.1, Control 26.7 ± 3.7.	Dietary intake (7DFR and NU-AGE Index)	↑ NU-AGE Index (p<0.01)<br>↑ wholegrains (g/day) (p<0.01)<br>↑ fruits (g/day) (p=0.02)<br>↑ vegetables (g/day) (p<0.01)<br>↑ legumes (g/day) (p<0.01)<br>↑ low-fat dairy (g/day) (p<0.01)<br>↑ fish (g/day) (p<0.01)<br>↑ nuts (g/day) (p<0.01)

								↑ olive oil (g/day) (p<0.01)<br>↓ alcohol (g/day) (p=0.02)<br>↓ sodium (mg/day) (p=<0.01)<br>↓ sweets (g/day) (p<0.01)
Bihuniak et al. (2016), USA	Pre/post	Postmenopausal women	To assess the efficacy of a MD intervention in a group of postmenopausal women in the USA and examine the influence of a MD on cardiovascular risk factors	6 months	N/A	Sample size: n = 22. Retention: 72.7%. Age: 77 ± 6.8. BMI: 25.4 ± 2.9.	MDS, dietary intake (3DFR), TC, LDL-c, HDL-c, fatty acids	↑ MDS (p<0.001)<br>↑ fat (g and %E) (p<0.05)<br>↑ omega-3 fatty acids (g and %E) (p<0.01)<br>↑ omega-6 fatty acids (g and %E) (p<0.01)<br>↑ HDL-c (p<0.05)<br>↑ EPA (p<0.01)<br>↑ DHA (p<0.001)<br>↓ %E CHO (p<0.05)<br>↓ sugar (g) (p<0.05)<br>↓ TG (p<0.001)<br>≠ SFA (%E and g), fibre, TC, LDL-c
Choi et al. (2019), USA	RCT, parallel	CVD	To evaluate the effectiveness of dietary counselling provided through a custom smartphone app for the implementation and compliance to a MD	3 months	3 months (6 months since baseline)	Sample size: n = 100 (Intervention n = 51, Control n = 49). Retention: 89% (Intervention 88.2%, Control 88.8%). Age: Intervention 57.2 ± 1.8, Control 56.6 ± 1.7. Sex: Intervention 22F (43.1%), Control 17F (34.7%). BMI: Intervention 29.5 ± 0.6, Control 30.8 ± 0.6.	MDS, hs-CRP, weight, BP, TC, TG, LDL-c, HDL-c	<u>Post-intervention:</u><br>↑ MDS (p>0.05)[c]<br>↓ weight (p<0.05)<br>≠ MDS, hs-CRP, BP, TC, TG, LDL-c, HDL-c (all p>0.05)<br><br><u>Follow-up:</u><br>↑ MDS (p<0.001)[c]<br>↓ weight (p=0.04)<br>≠ MDS, hs-CRP, BP, TC, TG, LDL-

								c, HDL-c (all p>0.05)
Chou et al. (2022), Taiwan	RCT, parallel	Healthy older adults	To investigate the effect of a mini-flipped, game-based MD learning program on dietary behaviour and cognitive function	8 weeks	N/A	Sample size: n = 84 (Intervention n = 43, Control n = 41). Retention: 95.2% (Intervention 95.3%, Control 95.1%). Sex: Intervention 38F (92.7%), Control 29F (74.4%). BMI: Intervention 24.35 ± 3.56, Control 23.99 ± 3.80.	MDS[h]	↑ MDS (p<0.001)
Cooper et al. (2022), Australia	Pre/post	Adults with knee osteoarthritis	To determine the feasibility of an anti-inflammatory diet intervention delivered via telehealth for patients with knee osteoarthritis	9 weeks	N/A	Sample size: n = 28. Retention: 78.6%. Age: 66 ± 8. Sex: 22F (82%). BMI: 30.6 ± 4.6.	Dietary intake(24hr recall and 3DFR)	↑ total fat (%E and g) (p<0.05)<br>↑ PUFA (g) (p=0.019)<br>↑ MUFA (g) (p=0.003)<br>↓ CHO (%E and g) (p<0.05)<br>≠ SFA, fibre, vegetables, fruit, wholegrains
Davis et al. (2017); Murphy et al. (2022), Australia	RCT, parallel	Healthy older adults	To determine how well healthy older adults in Australia can adopt the MD	6 months	12 months (18 months since baseline)	Sample size: n = 166 (Intervention n = 85, Control n = 81). Retention: Age: Intervention 71.0 ± 4.9, Control 70.9 ± 4.9. Sex: Intervention 57.5%F, Control 55.1%F. BMI: Intervention 26.7 ± 3.7, Control 27.1 ± 4.1.	MDS[d], dietary intake (3DFR), serum fatty acids	<u>Post-intervention:</u><br>↑ MDS (p<0.01)<br>↑ EVOO (p<0.001)<br>↑ vegetables (p<0.001)<br>↑ fruit (p<0.001)<br>↑ nuts (p<0.001)<br>↑ legumes (p<0.001)<br>↑ dairy (p<0.001)<br>↑ fish/seafood (p<0.001)<br>↑ %E fat (p<0.01)<br>↑ %E MUFA (p<0.001)<br>↑ MUFA:SFA ratio (p<0.001)

								↑ fibre (p<0.001)<br>↑ omega-3 fatty acids (p<0.001)<br>↑ erythrocyte MUFA (p<0.001)<br>↑ DHA (p=0.01)<br>↓ meats (p=0.03)<br>↓ miscellaneous (p<0.001)<br>↓ %E SFA (p<0.001)<br>↓ %E CHO (p<0.001)<br>↓ sugars (p<0.01)<br>↓ sodium (p<0.001)<br>↓ erythrocyte SFA (p<0.001)<br><br><u>Follow-up:</u>[e]<br>↓ MEDAS (p<.001)<br>↓ legumes (p<.001)<br>↓ vegetables (p=0.03)<br>↓ breads (p<.001)<br>↑ alcohol (p=0.03)<br>↑ discretionary foods (p<.001)<br>↑ SFA (g/MJ/day) (p<.001)
Droste et al. (2013a); (2013b), Luxembourg	RCT, parallel	Arteriosclerosis	To assess the effect of lifestyle counselling on lipid profile in patients with arteriosclerosis	4 weeks	16 weeks (20 weeks since baseline)	Sample size: n = 122 (Intervention n = 60, Control n = 62). Retention: Intervention 88.3%, Control 88.7%. Age: Intervention 63.7 ± 8.1, Control 63.4 ± 10.6. Sex: Intervention 64%M, Control, 69%M. BMI:	Dietary intake (3DFR), BMI, TC, TG, LDL-c, HDL-c	<u>Post-intervention:</u><br>↑ PUFA (g) (p<0.001)<br>↑ fibre (g) (p<0.001)<br>↑ nuts (g) (p<0.001)<br>↑ vegetables (g) (p<0.001)

						Intervention 27.3 ± 4.5, Control 27.8 ± 4.2.		↑ tomatoes (p<0.001)<br>↓ energy intake (kcal) (p=0.026)<br>↓ CHO (g) (p=0.047)<br>↓ SFA (g) (p=0.019)<br>↓ BMI (p=0.025)<br><br><u>Follow-up:</u><br>↑ PUFA (g) (p<0.001)<br>↑ fibre (g) (p<0.001)<br>↑ nuts (g) (p<0.001)<br>↑ vegetables (g) (p<0.001)<br>↑ tomatoes (p<0.001)<br>↓ energy intake (kcal) (p=0.049)<br>↓ CHO (g) (p=0.037)<br>↓ TC (p=0.023)<br>↓ LDL-c (p=0.029)<br>↓ LDL:HDL ratio (p=0.024)<br>↓ TG (p=0.036)
Entwistle et al. (2018), UK	RCT, parallel	Thoracic transplant recipients	To assess adherence in thoracic transplant recipients to a dietary intervention known to reduce CVD risk factors	12 months	6 weeks	Sample size: n = 41 (Intervention n = 21, Control n = 20). Retention: Intervention 95.2%, Control 95%. Age: Intervention 58 [33-65], Control 59 [27-65]. Sex: Intervention 15M (71%), Control 14M (70%). BMI: Intervention 29 ± 4, Control 29 ± 5.	MDS, MDS Index[i], weight, BMI, TG	<u>Post-intervention:</u><br>↑ MDS (p<0.001)<br>↑ MDS Index (p<0.05)<br>↓ weight (p<0.05)<br>↓ BMI (p<0.05)<br>↓ TG (p<0.05)<br><br><u>Follow-up:</u><br>↑ MDS (p<0.001)

								↑ MDS Index (p<0.05)
Estruch et al. (2018), Spain	RCT, parallel	Adults at high cardiovascular risk	To assess the efficacy of two MDs (one supplemented with EVOO and one supplemented with nuts) on primary cardiovascular prevention	5 years	N/A	Sample size: n = 7447 (Med diet + EVOO n = 2543, Med diet + nuts n = 2454, Control n = 2450). Age: Med diet + EVOO 67.0 ± 6.2, Med diet + nuts 66.7 ± 6.1, Control 67.3 ± 6.3. Sex: Med diet + EVOO 1493F (58.7%), Med diet + nuts 1326F (54.0%), Control 1463F (59.7%). BMI: Med diet + EVOO 29.9 ± 3.7, Med diet + nuts 29.7 ± 3.8, Control 30.2 ± 4.0.	MEDAS, dietary intake (FFQ)	<u>MD + EVOO:</u><br>↑ MEDAS (p<0.001)<br>↑ EVOO (p<0.001)<br>↑ nuts (p<0.001)<br>↑ legumes (p<0.001)<br>↑ fish/seafood (p=0.01)<br>↑ %E fat (p<0.001)<br>↑ %E SFA (p=0.004).<br>↑ %E MUFA (p<0.001)<br>↑ %E PUFA (p<0.001)<br>↓ energy intake (p<0.001)<br><br><u>MD + Nuts:</u><br>↑ MEDAS (p<0.001)<br>↑ EVOO (p<0.001)<br>↑ nuts (p<0.001)<br>↑ legumes (p<0.001)<br>↑ fish/seafood (p=0.001)<br>↑ fibre (p<0.001)<br>↑ %E fat (p<0.001)<br>↑ %E MUFA (p<0.001)<br>↑ %E PUFA (p<0.001)<br>↓ energy intake (p<0.001)
Grimaldi et al. (2018), Italy	Non-randomised	Adults at high cardiovascular risk	To investigate the efficacy and durability of a 3-	3 months	9 months	Sample size: n = 116 (Intervention n = 71, Control n = 45).	Dietary intake (7DFR),	<u>Post-intervention:</u><br>↑ fibre (p<0.001)

	interventio n		month intensive dietary intervention on body weight and metabolic risk factors			Retention: Intervention 88.7%, Control 64.4%. Age: Intervention 59 ± 10, Control 56 ± 11. BMI: Intervention 32.3 ± 4.2, Control 32.6 ± 3.9.	weight, BMI, TC, HDL-c, LDL-c, TG	↓ SFA (g) ($p<0.001$)<br>↓ weight ($p<0.001$)<br>↓ HbA1c ($p<0.001$)<br>↓ triglycerides ($p<0.01$)<br>↓ BP ($p<0.01$)<br><br><u>Follow-up:</u><br>↑ fibre ($p<0.001$)<br>↓ energy (kcal) ($p<0.01$)<br>↓ total fat (%E and g) ($p<0.05$)<br>↓ SFA (%E and g) ($p<0.001$)<br>↓ weight ($p<0.001$)<br>↓ HbA1c ($p<0.001$)<br>↓ BP ($p<0.001$)
Hagfors et al. (2005), Sweden	RCT, parallel	Rheumatoid arthritis	To investigate the efficacy of a MD vs ordinary Western diet for suppression of disease activity and patients with rheumatoid arthritis	3 months	N/A	Sample size: n = 56 (Intervention n = 29, Control n = 27). Retention: Intervention 89.7%, Control 92.6%. Age: Intervention 58 [33-73], Control 59 [39.75]. Sex: Intervention 5M/21F, Control 5M/20F. BMI: Intervention 28.4 [21.5-38.7], Control 25.6 [16.4-31.4].	Dietary intake (self-report diet questionnaire and diet history interview)	↑ fish/seafood ($p<0.001$)<br>↑ poultry ($p<0.001$)<br>↑ low-fat cheese ($p=0.005$)<br>↑ seeds ($p=0.016$)<br>↑ total serum n-3 fatty acids ($p=0.001$)<br>↓ red and processed meat ($p<0.001$)<br>↓ full fat dairy ($p<0.001$)<br>↓ sweets ($p<0.001$)<br>↓ total serum n-6 fatty acids ($p=0.008$)

								↓ serum n-6:n-3 ratio (p=0.002)
Hardman et al. (2005), Australia	RCT, parallel	Healthy older adults	To investigate the effect of a change of diet to a MD and/or increase in walking-based exercise on cognition over 6-months	6 months	N/A	Sample size: n = 102 (Diet only n = 25, Diet and exercise n = 24, Control n = 27). Retention: Intervention 72%, Diet and exercise 75%, Control 92.6%. Age: Diet only 77.68 ± 7.38, Diet and exercise 76.54 ± 7.37, Control 78.22 ± 5.81. Sex; Diet only 20F, Diet and exercise 17F, Control 18F. BMI: Diet only 28.26 ± 5.37, Diet and exercise 26.64 ± 3.39, Control 29.01 ± 4.90.	MDS[j], dietary intake (FFQ), TC, hs-CRP, BP, HbA1c	Diet only:<br>≠ MDS, BP, TC, hs-CRP, HbA1c<br><br>Diet + exercise:<br>≠ MDS, BP, TC, hs-CRP, HbA1c
Katsarou et al. (2014), Greece	RCT, parallel	Hypertension	To examine the effect and feasibility of a combined intervention of dietary education and stress management on the control of hypertension	2 months	N/A	Sample size: n = 45 (Intervention n = 21, Control = 24). Retention: Intervention 76.2%, Control 83.3%. Age: Intervention 61.1 ± 10.9, Control 68.9 ± 10.7. Sex: Intervention 43.8%M, Control 35%M. BMI: Intervention 29.08 ± 4.30, Control 29.48 ± 4.41.	MDS[k], BP, BMI	≠ MDS, BP, BMI (all p>0.05)
Keyserling et al. (2016), USA	Pre/post	Healthy older adults	To develop and evaluate a dietary, physical activity and weight loss intervention for residents of south-eastern USA	6 months	N/A	Sample size: n = 339. Retention: 74%. Age: 56 ± 0.6. Sex: 260F (77%). BMI: 36 ± 0.5.	Dietary intake (DRA[l], FVS[m], DFQA[n]), weight, BP	↑ DRA (p<0.001)<br>↑ FV screener (p<0.01)<br>↑ DFQA (p<0.001)<br>↓ weight (p<0.01)<br>↓ BP (p<0.001)
Landaetea-Diaz et al.	RCT, parallel	Adults with MetS	To investigate how the different	3 months	N/A	Sample size: n = 45 (Diet only n = 24, Diet and	MEDAS, dietary	Diet only:

(2012), Spain			dimensions of health-related quality of life evolved in metabolic syndrome patients after following a hypocaloric MD			exercise n = 21). Retention: Intervention 83.3%, Control 95.2%. Age: Diet only 57.2 ± 0.98, Diet and exercise 59.05 ± 1.22. Sex: Diet only 15F (62.5%), Diet and exercise 13F (61.9%). BMI: Diet only 38.44 ± 1.46, Diet and exercise 37.05 ± 0.72.	intake (3DFR)	↑ MEDAS (p<0.05)<br>↑ MUFA (%E) (p<0.05)<br>↓ energy (kcal) (p<0.05)<br>↓ total fat (%E) (p<0.05)<br>↓ SFA (%E) (p<0.05)<br>↓ weight (p<0.05)<br>↓ BP (p<0.05)<br>↓ total cholesterol (p<0.05)<br><br><u>Diet + exercise:</u><br>↑ MEDAS (p<0.05)<br>↑ MUFA (%E) (p<0.05)<br>↓ energy (kcal) (p<0.05)<br>↓ total fat (%E) (p<0.05)<br>↓ SFA (%E) (p<0.05)<br>↓ BP (p<0.05)<br>↓ total cholesterol (p<0.05)
Maher et al. (2020), Australia	Pre/post	Healthy older adults	To assess the preliminary effect of a virtual health coach for improving physical activity, MD adherence and health risk factors	3 months	N/A	Sample size: n = 31. Retention: 90.3%. Age: 56.2 ± 8.0. Sex: 18F (64.3%).	MEDAS, weight, BP	↑ MEDAS (p<0.001)<br>↓ weight (p=0.01)<br>↓ waist circumference (p=0.003)<br>≠ BP
Marcos-Forniol et al.	RCT, parallel	ACS	To evaluate the efficacy of a secondary CHD	12 months	N/A	Sample size: n = 127 (Intervention n = 64, Control n = 63).	MDS[j], dietary intake (FFQ),	↑ MDS (p=0.013)<br>↓ BP (p<0.05)<br>↓ LDL-c (p<0.05)

(2018), Spain			prevention programme on risk factor control, adherence to a MD, quality of life, physical function, readmission and mortality in elderly patients with a recent coronary event			Retention: Intervention 84.4%, Control 82.5%. Age: Intervention 76 [74.1-79.2], Control 75.4 [73.7-78.9]. Sex Intervention 17F (31.5%), Control 25F (48.1%).	BMI, BP, LDL-c, HbA1c	↓ HbA1c ($p<0.05$)<br>≠ BMI ($p>0.05$)
Martinez-Rodriguez et al. (2021), Spain	RCT, parallel	Healthy older women	To evaluate the effect of the addition of aquatic resistance interval training to a nutritional intervention on body composition, body image perception and adherence to the MD	14 weeks	N/A	Sample size: n = 40 (Intervention n = 20, Control n = 20). Retention: 85%. Age: Intervention 69.6 ± 5.0, Control 67.7 ± 3.6. BMI: Intervention 28.8 ± 4.7, Control 28.2 ± 4.2.	MEDAS	≠ MEDAS ($p>0.05$)
Masumi et al. (2022), Iran	RCT, parallel	Healthy older adults	To examine the effectiveness of small group education on nutritional knowledge and attitudes towards the MD	4 weeks	N/A	Sample size: n = 100 (Intervention n = 50, Control n = 50). Retention: 91% (Intervention 92%, Control 90%). Age: Intervention 63.1 ± 3.7, Control 62.3 ± 3.9.	MEDAS[g], BMI and BP	↑ MEDAS ($p<0.001$)<br>≠ BMI, BP
Mayr et al. (2019), Australia	RCT, parallel	CHD	To evaluate the efficacy of a 6-month MD intervention in reducing the incidence of secondary CVD	6 months	6 months (12 months since baseline)	Sample size: n = 73 (Intervention n = 37, Control n = 36). Retention: Intervention 73.0%, Control 80.6%. Age: Intervention 61.8 ± 9.2, Control 61.8 ± 9.5.	MEDAS, dietary intake (7DFR)	<u>Post-intervention:</u><br>↑ MEDAS ($p<0.001$)<br>↑ vegetable, tomato ($p<0.05$))<br>↑ yoghurt ($p<0.05$))

			events compared with patients on a low-fat diet			Sex: Intervention 27M (79.4%), Control 27M (87.1%). BMI: Intervention 30.7 ± 5, Control 29.1 ± 5.3.		↑ nuts ($p<0.05$)<br>↑ legumes ($p<0.05$)<br>↑ seafood ($p<0.05$)<br>↑ unsaturated oils ($p<0.05$)<br>↑ olive oil ($p<0.05$)<br>↑ MUFA (%E) ($p<0.05$)<br>↑ PUFA (%E) ($p<0.05$)<br>↑ n-3 (g) ($p<0.05$)<br>↓ processed meat ($p<0.05$)<br>↓ CHO (%E) ($p<0.05$)<br><br>Follow-up:[e]<br>↓ MEDAS ($p=0.01$)
McGrattan et al. (2021) Ireland	RCT, parallel	MCI	To evaluate the feasibility, acceptability and implementation of the THINK-MED intervention among older adults with cognitive impairment	6 months	6 months (12 months since baseline)	Sample size: n = 15 (THINK-MED baseline n = 5, THINK-MED staged n = 4, Control n = 6). Retention (post-intervention): 60% (THINK-MED baseline 60%, THINK-MED staged 75%, Control 50%); (12-month follow-up): 40% (THINK-MED baseline 60%, THINK-MED staged 25%, Control 33%). Age: THINK-MED staged 73.7 ± 12.1, THINK-MED baseline 79.2 ± 5.0, Control 75.7 ± 6.0. Sex: THINK-MED staged 3M (20.0%),	MDS	THINK-MED staged:[c,f]<br>↑ MDS<br><br>THINK-MED baseline:[c,f]<br>↑ MDS

						THINK-MED baseline 4M (26.7%), Control 2M (13.3%). BMI: THINK-MED staged 28.6 ± 4.8, THINK-MED baseline 26.0 ± 3.2, Control 29.1 ± 4.1.		
Michaelsen et al. (2006), Germany	RCT, parallel	CAD	To investigate the effect of a MD on markers of inflammation and metabolic risk factors in patients with treated CAD	12 months	N/A	Sample size: n = 105 (Intervention n = 51, Control n = 54. Retention: Intervention 94.1%, Control 98.1%. Age: Intervention 59.0 ± 8.7, Control 59.8 ± 8.6. Sex: Intervention 79.2%M, Control 75.5%M. BMI: Intervention 26.1 ± 3.2, Control 27 ± 2.8.	Dietary intake (7DFR), plasma fatty acids, hs-CRP, TC, LDL-c, HDL-c, TG, fasting insulin, BMI	↑ fruits ($p=0.038$)<br>↑ low-fat dairy products ($p<0.010$)<br>↑ wholegrain bread/pasta ($p<0.010$)<br>↑ fish ($p<0.010$)<br>↑ walnuts ($p<0.010$)<br>↑ EPA ($p=0.026$)<br>↑ DHA ($p=0.022$)<br>↓ meat/sausages ($p<0.010$)<br>↓ %E CHO ($p=0.034$)<br>↓ %E total fat ($p=0.011$)<br>↓ %E SFA ($p<0.001$)<br>↓ %E MUFA ($p=0.024$)<br>≠ vegetables ($p=0.067$)<br>≠ cakes/sweets ($p=0.646$)<br>≠ fibre ($p=0.399$)<br>≠ BMI, hs-CRP, TC, LDL-c, HDL-c, TG, fasting insulin (all $p>0.05$)
Quintana-Navarro et al. (2020), Spain	RCT, parallel	CAD	To investigate the dietary changes and assess both the level of	5 years	N/A	Sample size: n = 1002 (Intervention n = 502, Control n = 500. Retention: Intervention	MEDAS, dietary intake (FFQ)	↑ MEDAS ($p<0.001$)<br>↑ %E EVOO ($p<0.05$)

			adherence and long-term maintenance following a MD versus a low-fat diet			89%, Control 81.2%. Age: Intervention 59.7 ± 0.4, Control 59.5 ± 0.4. Sex: Intervention 414M (82.5%), Control 413M (82.6%). BMI: Intervention 31.0 ± 0.1, Control 31.2 ± 0.2.		↑ %E fish/seafood ($p<0.05$)<br>↑ %E nuts ($p<0.05$)<br>↓ %E red/processed meat ($p<0.05$)<br>↓ %E cereals ($p<0.05$)<br>↓ %E commercial bakery products ($p<0.05$)<br>↓ %E dairy products ($p<0.05$)<br>↓ %E seed oils ($p<0.05$)<br>≠ %E vegetables, fruits, legumes, sweet/carbonated drinks (all $p>0.05$)
Rusch et al. (2021), USA	Pre/post	Parkinson's disease	To determine whether adhering to the MD is feasible and induces beneficial changes in gastrointestinal function, intestinal permeability and faecal microbial communities in individuals with Parkinson's disease	5 weeks	N/A	Sample size: n = 8. Retention: 100%. Age: 71.4 ± 2.6. Sex: 5M (63%). BMI: 26.7 ± 1.4.	MEDAS, dietary intake (24hr recall)	↑ MEDAS ($p<0.001$)<br>↑ fibre ($p=0.027$)<br>↑ fat (g and %E) ($p<0.05$)<br>↑ MUFA (g and %E) ($p<0.05$)<br>↑ PUFA (%E) ($p=0.009$)<br>↓ CHO (%E) ($p=0.036$)
Salas-Salvado et al. (2019), Spain	RCT, parallel	Overweight/obese adults with MetS	To evaluate the effect of an intensive weight loss intervention based on an	12 months	N/A	Sample size: n = 698 (Intervention n = 361, Control n = 337). Retention: Intervention 88.1%, Control 86.6%.	Dietary intake (MD questionnaire and FFQ)	↑ MD questionnaire ($p<0.001$)<br>↑ MUFA (%E) ($p<0.001$)

			energy-restricted MD, physical activity and behaviour support on hard cardiovascular events compared to usual care			Age: Intervention 66 ± 5, Control 65 ± 5. Sex: Intervention 148M (45%), Control 141M (47%). BMI: Intervention 32.3 ± 3.4, Control 32.6 ± 3.6.		↑ nuts (g/day) ($p=0.05$) ↑ wholegrain cereals ($p=0.01$) ↓ refined cereals ($p<0.001$)
Schwartz et al. (2019), USA	RCT, parallel	Cognitive decline (mild dementia, MCI, SCD)	To investigate the efficacy of a behaviour health coaching intervention to increase adherence to behaviours associated with brain health and improve quality of life in patients at risk for cognitive decline	6 months	N/A	Sample size: n = 40 (Intervention n = 21, Control n = 19). Retention: 92.5%. Age: Intervention 74.7 ± 10.4, Control 74.3 ± 8.0. Sex: Intervention 10M/9F, Control 6M/12F. BMI: Not described.	MedScore (calculated from FFQ)	↑ MedScore ($p=0.016$)
Toobert et al. (2005), USA	RCT, parallel	Postmenopausal women with T2D	To evaluate the effects of a Mediterranean lifestyle program versus usual care on behaviour risk factors for CHD in women with T2D	6 months	N/A	Sample size: n = 279 (Intervention n = 163, Control n = 116). Retention: 88%. Age: Intervention 61.1 ± 8.0, Control 60.7 ± 7.8. BMI: Intervention 35.1 ± 7.7, Control 35.6 ± 8.8.	Dietary intake (FFQ), weight	↑ fruit ($p<0.001$) ↑ vegetables ($p<0.001$) ↓ total fat ($p<0.001$) ↓ SFA ($p<0.001$) ↓ weight ($p=0.004$)
Tuttle et al. (2008), USA	RCT, parallel	MI	To compare a low-fat versus MD on cardiovascular events and survival after first MI	2 years	N/A	Sample size: n = 101 (Intervention n = 51, Control n = 50). Retention: Intervention 72.5%, Control 72%.Age: Intervention 58 ± 10, Control 57 ± 10. Sex: Intervention 41M (80%), Control 75M (74%). BMI:	Dietary intake (3DFR), hs-CRP, weight, BMI, BP, TG, LDL-c, HDL-c, glucose, insulin	↑ omega-3 fats ($p<0.001$).

Zuniga et al. (2019), USA	RCT, parallel	Breast cancer survivors	To improve adherence to an anti-inflammatory dietary pattern in breast cancer survivors	6 months	N/A	Intervention 30 ± 5, Control 29 ± 6. Sample size: n = 153 (Intervention n = 76, Control n = 77). Retention: Intervention 78.9%, Control 84.4%). Age: Intervention 55.3 ± 10.3, Control 58.4 ± 8.2. BMI: Intervention 31.2 ± 4.1, Control 32.7 ± 5.2.	MEDAS, dietary intake (3DFR)	↑ MEDAS ($p<0.001$) ↓ energy intake ($p=0.045$) ≠ %E fat, %E SFA, fibre, fruit, vegetables

[a] Time since end of intervention. [b] If no control group, compared to baseline. [c] Compared to baseline. [d] Dietary outcomes are assessed at 4 months. [e] Compared to post-intervention. [f] Study was under-powered and did not test for significance. [g] MEDAS (question on wine intake removed, scores ranging from 0-13). [h] MDS (Chinese version, scores ranging from 0-17). [I] MDS Index (scores from the MDS were rescaled to range from 0-100 to reflect percentage of score achieved). [j] MDS (scores ranging from 0-9). ↑ or ↓ indicates the intervention significantly improved outcomes compared to control and ≠ indicates no significant difference between intervention and control groups. [k]MDS (scores ranging from 0-55). [l]DRA (scores ranging from 0-52, higher scores indicate higher diet quality). [m]FVS (scores rangings from 0-35, higher scores indicate greater intake from fruit/vegetables). [n]DFQA (scores ranging from 0-24, higher scores indicate greater healthful fat intake).

Abbreviations: %E, percentage of energy; 3DFR, 3-day food record; 7DFR, 7-day food record; ACS, acute coronary syndrome; BMI, body mass index; BP, blood pressure; CAD, coronary artery disease; CHD, coronary heart disease; CHO, carbohydrates; CRF, chronic renal failure; DFQA, dietary fat quality assessment; DHA, docosahexaenoic acid; DRA, dietary risk assessment; EPA, eicosapentaenoic acid; EVOO, extra virgin olive oil; FFQ, food frequency questionnaire; FVS, fruit and vegetable screener; HDL-c, high-density lipoprotein cholesterol; hs-CRP, high sensitivity c-reactive protein; IL, interleukin; LDL-c, low-density lipoprotein cholesterol; MCI, mild cognitive impairment; MD, Mediterranean diet; MDS, Mediterranean Diet Score; MEDAS, MEditerranean Diet Adherence Screener; MetS, metabolic syndrome; MI, myocardial infarction; MUFA, monounsaturated fat; PUFA, polyunsaturated fat; RCT, randomised controlled trial; s/d, servings per day; s/w, servings per week; SCD, subjective cognitive decline; SFA, saturated fat; T2D, type 2 diabetes; TC, total cholesterol; TG, triglycerides; UK, United Kingdom; USA, United States of America; WDI, Wollongong Dietary Inventory.
