# Supplementary material for: Behaviour Change Techniques Used in Mediterranean Diet Interventions for Older Adults: A Systematic Scoping Review
Source: Nutrients. 2023 Feb 27;15(5):1189. doi: 10.3390/nu15051189 (PMC10005068; doi:10.3390/nu15051189)
Supplement: Supplementary file 1 [file nutrients-15-01189-s001.zip › Nutrients_Table S3_BCT definitions.pdf]

Table S3. Definitions of identified behaviour change techniques from the Behaviour Change Technique Taxonomy (version 1) *

Label	Definition
**1. Goals and planning**	
1.1 Goal setting (behaviour)	Set or agree on a goal defined in terms of the behaviour to be achieved
1.2 Problem solving	Analyse , or prompt the person to analyse, factors influencing the behaviour and generate or select strategies that include overcoming barriers and/or increasing facilitators
1.3 Goal setting (outcome)	Set or agree on a goal defined in terms of a positive outcome of wanted behaviour
1.5 Review behaviour goal(s)	Review behaviour goal(s) jointly with the person and consider modifying goal(s) or behaviour change strategy in light of achievement. This may lead to re-setting the same goal, a small change in that goal or setting a new goal instead of (or in addition to) the first, or no change
1.6 Discrepancy between current behaviour and goal	Draw attention to discrepancies between a person's current behaviour (in terms of the form, frequency, duration, or intensity of that behaviour) and the person's previously set outcome goals, behavioural goals or action plans (goes beyond self-monitoring of behaviour)
1.7 Review outcome goal(s)	Review outcome goal(s) jointly with the person and consider modifying goal(s) in light of achievement. This may lead to resetting the same goal, a small change in that goal or setting a new goal instead of, or in addition to the first
1.8 Behavioural contract	Create a written specification of the behaviour to be performed, agreed on by the person, and witnessed by another
**2. Feedback and monitoring**	
2.2 Feedback on behaviour	Monitor and provide informative or evaluative feedback on performance of the behaviour
2.3 Self-monitoring of behaviour	Establish a method for the person to monitor and record their behaviour(s) as part of a behaviour change strategy
2.4 Self-monitoring of outcome(s) of behaviour	Establish a method for the person to monitor and record the outcome(s) of their behaviour as part of a behaviour change strategy

**3. Social support**	
3.1 Social support (unspecified)	Advise on, arrange or provide social support (e.g. from friends, relatives, colleagues,' buddies' or staff) or noncontingent praise or reward for performance of the behaviour. It includes encouragement and counselling, but only when it is directed at the behaviour
**4. Shaping knowledge**	
4.1 Instruction on how to perform the behaviour	Advise or agree on how to perform the behaviour
**5. Natural consequences**	
5.1 Information about health consequences	Provide information (e.g. written, verbal, visual) about health consequences of performing the behaviour
**6. Comparison of behaviour**	
6.1 Demonstration of the behaviour	Provide an observable sample of the performance of the behaviour, directly in person or indirectly e.g. via film, pictures, for the person to aspire to or imitate
**8. Repetition and substitution**	
8.1 Behavioural practice/rehearsal	Prompt practice or rehearsal of the performance of the behaviour one or more times in a context or at a time when the performance may not be necessary, in order to increase habit and skill
**9. Comparison of outcomes**	
9.1 Credible source	Present verbal or visual communication from a credible source in favour of or against the behaviour
**10. Reward and threat**	
10.1 Material incentive (behaviour)	Inform that money, vouchers or other valued objects will be delivered if and only if there has been effort and/or progress in performing the behaviour
10.3 Non-specific reward	Arrange delivery of a reward if and only if there has been effort and/or progress in performing the behaviour

**12. Antecedents**	
12.5 Adding objects to the environment	Add objects to the environment in order to facilitate performance of the behaviour

* For the full Behaviour change technique taxonomy refer to the publication *"The Behavior Change Technique Taxonomy (v1) of 93 Hierarchically Clustered Techniques: Building an International Consensus for the Reporting of Behavior Change Interventions"* by Michie et al (2013).
